# Impact of disease-modifying therapy on dendritic cells and exploring their immunotherapeutic potential in multiple sclerosis

**DOI:** 10.1186/s12974-022-02663-z

**Published:** 2022-12-12

**Authors:** Caiyun Liu, Jie Zhu, Yan Mi, Tao Jin

**Affiliations:** 1 Neuroscience Center, Department of Neurology, The First Hospital of Jilin University, Changchun, China; 2 Department of Neurobiology, Care Sciences & Society, Division of Neurogeriatrcs, Karolinska Institutet, Karolinska University Hospital Solna, Stockholm, Sweden

**Keywords:** Multiple sclerosis, Immunotherapy, Tolerance, Dendritic cell

## Abstract

Dendritic cells (DCs) are the most potent professional antigen-presenting cells (APCs), which play a pivotal role in inducing either inflammatory or tolerogenic response based on their subtypes and environmental signals. Emerging evidence indicates that DCs are critical for initiation and progression of autoimmune diseases, including multiple sclerosis (MS). Current disease-modifying therapies (DMT) for MS can significantly affect DCs’ functions. However, the study on the impact of DMT on DCs is rare, unlike T and B lymphocytes that are the most commonly discussed targets of these therapies. Induction of tolerogenic DCs (tolDCs) with powerful therapeutic potential has been well-established to combat autoimmune responses in laboratory models and early clinical trials. In contrast to in vitro tolDC induction, in vivo elicitation by specifically targeting multiple cell-surface receptors has shown greater promise with more advantages. Here, we summarize the role of DCs in governing immune tolerance and in the process of initiating and perpetuating MS as well as the effects of current DMT drugs on DCs. We then highlight the most promising cell-surface receptors expressed on DCs currently being explored as the viable pharmacological targets through antigen delivery to generate tolDCs in vivo.

## Introduction

Multiple sclerosis (MS) is a chronic inflammatory autoimmune disease of the central nervous system (CNS) pathologically featured by multi-spatiotemporal demyelination as well as neuronal and axonal degeneration and damage [1], affecting physical functions, cognition, quality of life, and employment. The underlying mechanisms of this disease remain to be unraveled. Immune tolerance invalidation triggered by various environmental factors (e.g., tobacco smoking, Epstein–Barr virus infection, ambient ultraviolet radiation, and vitamin D levels) in the context of genetic susceptibility [e.g., major histocompatibility complex (MHC) HLA-DRB1 locus] is thought to play a central role [2–4]. Under physiological conditions, most autoreactive T or B cells are deleted through central tolerance mechanisms in the primary lymphoid organs, such as thymus and bone marrow, while those that have escaped central tolerance will be counteracted through peripheral tolerance in the secondary lymphoid organs (including lymph nodes, spleen and mucosal-associated lymphoid tissues) as well as peripheral tissues [5]. As the immune tolerance has been broken in MS, CNS-directed autoreactive T and B cells can be activated in the periphery via molecular mimicry, CNS-releasing myelin protein-derived antigens, novel autoantigen or bystander activation [6]. Then, innate immune cells, cluster of differentiation (CD)8^+^ cytotoxic T cells, CD4^+^ T helper 1 (T_H_1) and T_H_17 cells, and B cells infiltrate into the CNS parenchyma and induce an inflammatory cascade, leading to demyelination, axonal loss and brain atrophy [6]. Emerging evidence has indicated that dendritic cells (DCs) act as a pivotal cell type governing the immune tolerance [7], and play a critical role in the initiation and perpetuation of MS [8, 9]. Given the unique ability of DCs to coordinate innate and adaptive immunity as well as the dual capacity to induce either inflammatory or tolerogenic responses depending on their subtypes and environmental signals, emerging therapies targeting or exploiting DCs have been being considered to combat autoimmune responses [10, 11]. In the present review, we summarized the role of DCs in governing immune tolerance and initiating and perpetuating MS as well as the effects of current disease-modifying therapies (DMT) on DCs. What is more, we focus on the potential therapeutic value of inducing tolDCs in MS therapy with emphasis on the promising surface receptors expressed on DCs currently being explored as the potential viable targets.

## DCs as the pivotal cells governing immune tolerance

### Human DC subsets and DC activation status

Human DCs constitute a phenotypically and functionally heterologous population of antigen-presenting cells (APCs) [11–17], which are generally divided into four major subsets based on developmental origin, specific surface markers and transcriptome profiles: type 1 conventional DC subset (cDC1), type 2 conventional DC subset (cDC2), plasmacytoid DC subset (pDC) and monocyte-derived DC subset (moDC) [18]. The features of human DC subsets with different locations, surface receptors and cytokine profiles as well as various functions are presented in Table 1. Different from the relative homogeneity of the cDC1 subset, the cDC2 subset can be further subdivided into two principal lineages defined as cDC2A and cDC2B lineages according to distinguishing developmental origins regulated by the transcription factors T-bet and RORγt, respectively [19]. The cDC2B lineage is more prone to secreting pro-inflammatory cytokines than the cDC2A lineage, while both are potent stimulators to prime CD4^+^ T cells [19]. In addition, several single-cell RNA-sequencing studies have reported the heterogeneity of cDC2s [18, 20]. Most recently, Ginhoux et al*.* proposed to define CD5^−^CD163^+^CD14^+^ cDC2 population as “DC3s”, a new DC subset that expands in inflammatory state independently of cDC1/cDC2 lineage [21]. However, further research is needed to better define the distinct differentiation pathways and molecular states occurring in different inflammatory environments.

**Table 1:** Characteristics of surface receptors of DC subsets in humans

Human DC subsets	cDC1	cDC2	pDC	moDC	Ref.
Subdivided lineages	Homogeneous	cDC2A and cDC2B			[18, 19]
Murine equivalent	CD8α^+^ cDC	CD11b^+^ cDC	Murine pDC		[12, 17]
Location	Bone marrow, blood, lymph nodes, spleen, tonsil and non‐lymphoid tissues	Blood, lymphoid organs and peripheral tissues	Bone marrow, blood and peripheral lymphoid tissues	Recruited from the bone marrow to inflamed tissues	[11–14]
Surface receptors	CD11c CD141 (BDCA3) CD205 (DEC205) DNGR-1 (CLEC9A) CD371 (CLEC12A, MICL) HLA-DR XCR-1 NECL2 TLR3, TLR9 and TLR10	CD11c CD1c (BDCA1) CD11b CD172a (SIRP-α) DCIR (CLEC4A) CD301a (CLEC10A) CD371 (CLEC12A, MICL) Langerin^low^ HLA-DR All TLRs excerpt TLR9	CD11c^low/−^ CD123 CD303 (BDCA2) CD304 (BDCA4) CD371 (CLEC12A, MICL) HLA-DR TLRs (7, 9, 10)	CD11c CD1a CD1c (BDCA1) CD14 CD64 (FcγRI) CD172a (SIRP-α) CD206 (MR) CD209 (DC-SIGN) FcεRI HLA-DR TLRs (1, 2, 3, 4, 5, 8, 9)	[12–14, 16, 17]
Cytokine profiles	IFN-III, IL-12, TNF-α, CXCL9, CXCL10	IL-1, IL-8, IL-10, IL-12, IL-23, TNF-α	IFN-I and III, TNF‐α, IL-6, Granzyme B	IL-1, IL-6, IL-10, IL‐12, IL-23, TNF‐α	[14, 15]
Functions	Antigen uptake and presentation, cross‐presentation, promoting T_H_1 and NK responses, priming CD8^+^ T cells	Antigen uptake and presentation, cross‐presentation, inducing polarization of T_H_1, T_H_2, T_H_17 cells, priming CD8^+^ T cells	Priming NK cells	Inducing pro-inflammatory responses during inflammation	[11, 13–15]

*CD* cluster of differentiation, *CLEC9A* C-type lectin domain family 9-member A, *DCIR* dendritic cell immunoreceptor, *DC-SIGN* dendritic cell-specific intercellular adhesion molecule-3-grabbing non-integrin, *DEC205* dendritic cell receptor for endocytosis-205, *DNGR-1* dendritic cell natural killer lectin group receptor-1, *FcεRI* the high affinity receptor for immunoglobulin E, *FcγRI* the high-affinity receptor for immunoglobulin G, *HLA-DR* human leukocyte antigen DR, *IFN* interferon, *IL* interleukin, *MICL* myeloid inhibitory C-type lectin receptor, *MR* mannose receptor, *SIRP-α* signal regulatory protein alpha, *T*_*H*_ T helper cells, *TLR* Toll-like receptor, *TNF‐α* tumor necrosis factor‐alpha, *XCR-1* chemokine XC receptor-1

During homeostatic conditions, most DCs reside throughout the body in a so-called immature phenotype, constantly surveying the surroundings by continually capturing and processing nearby environmental signals, including endogenous autoantigens and foreign microbial antigens [22]. Maturing DCs undergo various changes, including: (a) the processing of phagocytosis antigens; (b) the elevated presentation of antigens on MHC class I and II molecules; (c) the increased expression of critical co-stimulatory molecules, such as CD40, CD80, and CD86; (d) the enhanced secretion of polarizing cytokines, such as interleukin (IL)-6, IL-12, tumor necrosis factor (TNF)-α; (e) the up-regulation of chemokine receptor expression, such as CCR7 [12, 22]. DCs specifically present antigens to T cells via MHC-I or II molecules, whereas the downstream reaction of antigen recognition depends on the balance of signaling via co-receptors and cytokines which are associated with diverse stimuli received by DCs [11].

Tolerogenic DCs (tolDCs), another activated status of DCs, had been generally previously viewed to undergo incomplete maturation with limited changes in gene expression [23]; however, it has been overturned by a novel finding via genome-wide transcriptome analysis, that the transcriptome changes occurring during tolDC maturation were as complex as and largely overlapping with those occurring during immunogenic DC maturation [24]. In general, the tolDCs have the features with low levels of MHC and co-stimulatory molecules as well as pro-inflammatory cytokines, and expression of immunosuppressive molecules, such as inhibitory co-receptors (inducible co-stimulatory molecular ligand, programmed cell death ligand (PD-L)1 (B7-H1, CD274), PD-L2, B7-H3, and B7-H4), anti-inflammatory cytokines [IL-10 and transforming growth factor (TGF)-β], nitric oxide and indoleamine 2,3-dioxygenase (IDO) [25, 26].

Importantly, the tolDCs are capable of inducing regulatory T cells (Tregs), as well as quelling autoreactive CD4^+^ and CD8^+^ T cells in an antigen-specific manner [27], providing a precision therapeutic approach for autoimmune diseases. However, some researchers proposed that immunogenic and tolerogenic DCs might not be distinguished simply by the expression of pro-inflammatory genes, the presence of co-stimulatory molecules (CD80 and CD86) or TNF receptor family members [CD40, OX40 (CD134), and 4-1BB (CD137)], or by the absence of co-inhibitory molecules (e.g., PD-L1) [24]. This controversy challenges the induction and identification of tolDCs.

### The role of DCs in maintaining immune tolerance

Multiple natural tolerance mechanisms including central and peripheral tolerance perform a pivotal role in restraining the inappropriate immune responses [5]. Ohnmacht et al*.* reported that CD11c^+^ DC-depleted mice showed elevated frequency of CD4^+^ thymocytes as well as CD4^+^ T cell expansion and infiltration into peripheral tissues, resulting in a spontaneous fatal autoimmune disease featured by weight loss, splenomegaly, neutrophilia, T_H_1 and T_H_17 responses and autoantibody production [28]. Therefore, DCs are involved in implementing both tolerance processes and resolving ongoing immune responses by silencing and/or eliminating autoreactive T cells or inducing the generation and expansion of Tregs besides their roles in initiating and enhancing immunogenicity.

In the primary lymphoid organs, DCs maintain central immune tolerance by participating in negative selection of the autoreactive T cells. Both cDCs and pDCs in human thymus have been demonstrated to be capable of inducing the development of Tregs [29, 30]. A recent study identified a novel thymic CD14^+^Sirpα^+^ population of moDCs effectively recruited into the medulla region of thymus by medullary thymic epithelial cells in a Toll-like receptor (TLR)-dependent manner, conducing to the genesis of Tregs and thereby the establishment of central immune tolerance [31]. Interestingly, circulating DCs encountering many peripheral innocuous antigens can be recruited to the medulla region of thymus through an adhesion cascade [32].

Peripheral tolerance occurring in the secondary lymphoid organs provides a second layer of regulation, which is mainly mediated by peripheral DCs as well as other APCs [5]. In the absence of appropriate activation signals for instance co-stimulatory receptors and inflammatory cytokines, peripheral tolDCs capture, process and present endogenous antigens, eliciting the following T cell outcomes rather than inducing naïve T cell activation: (a) Deletion: autoreactive T cells undergo apoptosis mediated by “death receptors” on DCs such as Fas (CD95) and are deleted from the immune repertoire [11]; (b) Anergy: anergized T cells enter a nondividing, hyporeactive, and functionally inactive state, which is considered irreversible [33]; (c) Conversion into Tregs [34], exerting dominant immune suppression by secreting anti-inflammatory cytokines [35]. DCs induce peripheral T cell tolerance by various mechanisms, for example: (a) elevated production of anti-inflammatory cytokines (IL-10, IL-27, and TGF-β); (b) down-regulation of extracellular levels of adenosine and adenosine triphosphate; (c) enhanced synthesis of IDO inhibiting T cell proliferation accompanied by the ligation of CD80/CD86 on DCs with cytotoxic T-lymphocyte antigen 4 (CTLA-4) expressed on T cells; (d) expression of PD-L1 promoting T cell anergy; (e) up-regulated expression of TNF-related apoptosis-inducing ligand (TRAIL), CD95L and perforin inducing T cell deletion [26, 36].

In general, DCs are involved in implementing both central and peripheral tolerance processes. It is crucial and promising to elucidate the underlying mechanisms of DCs governing central and peripheral tolerance for exploring novel therapeutic targets of autoimmune diseases.

## The role of DCs in MS/experimental autoimmune encephalomyelitis (EAE)

It is recognized that DCs perform a continuous immune surveillance role in the healthy CNS, locating in vascular-rich regions, including perivascular spaces, meninges and choroid plexus [37]. During neuroinflammation, such as MS, DCs drastically accumulate in the CNS, invade the parenchyma, and prime myelin-specific T cell responses, contributing to the occurrence and development of MS [38, 39]. The inflammatory infiltration within MS lesions mainly consists of B cells, plasma cells, T cells, macrophages and DCs [40]. Once in the perivascular space, the DC-activated CD4^+^ T cells from the peripheral draining lymph nodes encounter CNS-derived antigen–MHC complexes presented by local APCs, followed by reactivation producing pro-inflammatory mediators, triggering infiltration of inflammatory cells [41]. Notably, cDCs, rather than other CNS-resident APCs including border-associated macrophages or microglia, have been demonstrated to be sufficient and essential for this process [37, 39, 42, 43]. A recent study reported that in cDCs, the nicotinamide adenine dinucleotide phosphate (NADPH) oxidase 2, also known as CYBB/NOX2, could modulate antigen processing and presentation and exert an essential role in the recruitment of encephalitogenic T_H_ cells during neuroinflammation [44].

Indeed, disease severity has been indicated to be related with the DC invasion into the CNS parenchyma in EAE, an animal model of MS. Sagar et al*.* found that DC transmigration measured by near-infrared imaging correlated with the severity of inflammation in EAE [45]. Furthermore, augmenting CNS-infiltrating DCs by intracerebral microinjection of stimulatory DCs [46] or by systemic treatment of recombinant FMS-like tyrosine kinase 3 ligand (Flt-3L): IgG [39] might exacerbate the onset and clinical disease of EAE. On the other hand, depletion of CD11c^+^ DCs could interfere with tolerance, leading to an enhanced inflammatory response and aggravated clinical course in murine EAE [34]. Similarly, selective depletion of CD11c^+^CD11b^+^ DCs and immature DCs but not CD11c^+^CD8^+^ DCs and mature DCs by injecting clodronate-loaded liposomes could partially, yet significantly, abrogate intravenous myelin oligodendrocyte glycoprotein (MOG)-induced suppression of EAE, implying that CD11c^+^CD11b^+^ DCs exert a tolerogenic effect [47].

In MS patients, the frequency and maturity of DCs may correlate with the disease stages and clinical patterns. Compared with myeloid DCs from healthy controls, those from relapsing–remitting (RRMS) or secondary-progressive MS (SPMS) had a higher proportion of CD40 expression. In addition, SPMS patients had an increased frequency of myeloid DCs expressing CD80 and those producing IL-12 and TNF-α, a decreased frequency of those expressing PD-L1 when compared with RRMS patients or healthy controls. In addition, the polarization effects on naïve T cells differed between myeloid DCs from patients with RRMS and SPMS [48]. Ectopic lymphoid follicles containing T cells, B cells, plasma cells and follicular DCs were detected along the meninges in SPMS patients [49]. DCs from patients with MS could secrete elevated amounts of pro-inflammatory cytokines, interferon (IFN)-γ, TNF-α, IL-6, IL-23 and IL-12p70, as well as increased levels of migratory molecules, such as CCR5 and CCR7, when compared to healthy individuals [50, 51]. However, pDCs from patients with MS expressed significantly low level of CD86 and 4-1BBL, compared with those from healthy controls and patients with a nonspecific inflammatory condition. When stimulated, pDCs from MS patients might manifest inefficient maturation features [52]. In addition, another study showed a significant reduction in the frequency of circulating pDCs in progressive MS patients [51]. These results suggest that DCs may be involved in the immunologic basis for the different clinical patterns of MS.

**Fig. 1:**
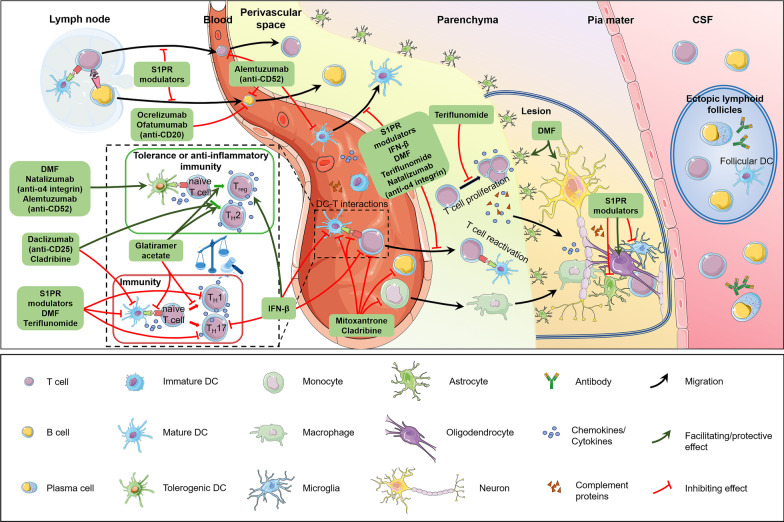
Roles of dendritic cells in the pathogenesis of multiple sclerosis and impacts of disease-modifying therapies. Immune tolerance invalidation is thought to play an important role in the pathogenesis of MS. DCs and B cells capture and process CNS-releasing myelin protein-derived antigens, migrate to secondary lymphoid organs and tissues, and present the self-antigens to T cells to induce myelin-reactive T_H_1 and T_H_17 cells. The activated T cells, B cells and DCs travel across the blood brain barrier (BBB). DCs drastically accumulate in the CNS, invade the parenchyma, and prime myelin-specific T cell responses. Once in the perivascular space, the DC-activated T cells from the peripheral draining lymph nodes encounter CNS-derived antigen–MHC complexes presented by local antigen-presenting cells (APCs) especially cDCs, followed by T cell reactivation. These autoreactive immune cells as well as pro-inflammatory mediators then migrate into CNS parenchyma. Ectopic lymphoid follicles containing T cells, B cells, plasma cells and follicular DCs are detected along the meninges in SPMS patients. The dual capacity of DCs to drive immunity and to confer antigen-specific T cell tolerance makes them a potential therapeutic target for MS by either inhibiting their immunogenicity or enhancing their tolerance. Current DMT drugs exert direct or indirect effects on DCs. IFN-β, sphingosine 1-phosphate receptor (S1PR) modulators, dimethyl fumarate (DMF), teriflunomide and glatiramer acetate reduce the polarization of pro-inflammatory T_H_1 and/or T_H_17 cells by influencing the maturation, antigen presentation and/or cytokine expression profiles of DCs. DMF, natalizumab and alemtuzumab are capable to induce tolerogenic DCs (tolDCs) conducing to the genesis of regulatory T cells (Tregs), thereby promoting the restoration of tolerogenic networks. Glatiramer acetate and IFN-β can also exert immunomodulatory effects through induction of Tregs. In addition, glatiramer acetate, daclizumab and cladribine might shift the cytokine synthesis pattern of human DCs toward an anti-inflammatory T_H_2 profile. Mitoxantrone and cladribine have non-specific effects, but also affect antigen presentation and cytokine production of DCs. S1PR modulators, IFN-β, DMF, teriflunomide and natalizumab can block the transit of DCs and autoreactive lymphocytes across the BBB. In the CNS, S1PR modulators exert beneficial effects on microgliosis and astrogliosis, alleviating demyelination. TolDCs, Tregs and T_H_2 cells generated in the periphery also modulate T_H_1 and T_H_17 cell production and microglial activity in the CNS

In conclusion, it is clear that peripherally derived DCs are essential for initiation and progression of MS/EAE (Fig. 1). The dual capacity of DCs to drive neuroinflammation and to confer antigen-specific T cell tolerance makes them a potential therapeutic target for MS by either inhibiting their immunogenicity or enhancing their tolerance. Indeed, various emerging immunotherapies targeting DCs have been developed for the treatment of MS.

## Impact of DMT on DCs

DCs play an important role in the pathogenesis of MS; however, these cells are rarely considered as targets for treatment in MS. Current DMT for MS may also affect DCs, even if T and B cells are the most frequently discussed targets for these therapies [53, 54]. The impacts of current DMT drugs on DCs are presented in Fig. 1.

### IFN‑β

The anti-inflammatory and regenerative effects of IFN-β are thought to underlie the efficacy of IFN-β in treating RRMS [55]. Diverse modes of action of IFN-β help contributing to its anti-inflammatory and immunoregulatory effects in MS, such as induction of autoreactive T cell apoptosis and inhibition of inflammatory cell migration across the blood–brain barrier (BBB), etc. [55, 56]. Notably, studies have indicated that IFN-β also performs several effects on DCs and other APCs.

First, IFN-β exerts both immunostimulatory and immunosuppressive effects on DCs. IFN-β could inhibit DC development at early stages but up-regulate the expression of co-stimulatory molecules on moDCs, enhancing the capacity of DCs to stimulate the secretion of IL-5, IL-10 and IL-13 by T cells [57]. In DCs derived from untreated MS patients and healthy controls, treatment with IFN-β-1a could up-regulate the gene expression of IL-12p35 and IL-27p28 and down-regulate the expression of IL-1β and IL-23p19 via signal transducer and activator of transcription (STAT)1 and STAT3 phosphorylation, respectively, consequently suppressing DC-mediated T_H_17 cell differentiation [58]. Furthermore, IFN-β^−/−^ DCs, when stimulated by lipopolysaccharide (LPS) or MOG peptide, might exhibit up-regulated expression of MHC-II and CD80 and secrete cytokines contributing to polarization of pathological T_H_17 cells rather than Tregs, compared with IFN-β^+/+^ DCs [59]. Genome-wide expression profiling and functional experiments showed that IFN-β non-responders had increased expression of co-stimulatory molecule CD86 on myeloid DCs before initiation of IFN-β therapy, implying that the clinical response to IFN-β may be related to the activation status of myeloid DCs [60], which is needed to elucidate the exact mechanisms of the nexus between activation status of myeloid DCs and action of IFN-β in MS/EAE in the future.

In addition to its general immunomodulatory actions, IFN-β could also inhibit the migratory capacity of DCs by down-regulating the production of migration-associated molecules [e.g., CCR7, matrix metalloproteinase (MMP)-9] via STAT1 signaling [61]. This inhibitory effect could be reversed when IFN-β was knocked out, further demonstrating the immunoregulatory role of IFN-β in the migration of DCs during EAE development [59]. With regard to MS-derived pDCs, IFN-β treatment could significantly down-regulate the TLR9 agonist-specific expression of CCR7, which was generally increased on pDCs from the untreated MS patients [62].

Finally, IFN-β-treated monocytes and DCs of MS patients increased expression of PD-L1, a potent inhibitor of autologous CD4^+^ T cell activation contributing to the maintenance of peripheral tolerance [63]. Besides, IFN-β could induce the apoptosis of mature DCs through the induction of caspase-11 expression and caspase-3 activation, which required the activation of STAT1 and NF-κB [64].

Interestingly, DC-targeted IFNs could increase the frequency of pDCs and induce their tolerogenic potential with up-regulated expression of IDO and TGF-β as well as induction of Tregs, effectively ameliorating EAE without significant side effects [65]. Thus, DC-specific targeted treatment may provide more efficient and safer therapy in MS.

### Glatiramer acetate

Glatiramer acetate is a synthetic random polymer composed of four amino acids including l-alanine, l-lysine, l-glutamic acid and l-tyrosine, structurally resembling myelin basic protein (MBP) [66]. It exerts anti-inflammatory and immunomodulatory effects and the best-documented mechanism of its effect is inducting T_H_2 cells and secreting anti-inflammatory cytokines, such as IL-4, IL-10 and TGF-β [53, 67], as well as inducting Tregs via activation of transcription factor FOXP3 [68]. Particularly, DCs are involved in the modes of action of glatiramer acetate in suppressing the induction of EAE and modulating the course of RRMS.

Glatiramer acetate promotes DC-mediated T cell differentiation shifting to T_H_2 polarization and Treg expansion [69]. In vitro studies have shown that glatiramer acetate can significantly suppress the proliferative effect of DCs on lymphocytes [70] and compete with MBP for binding to MHC molecules consequently inhibiting the antigen presentation process [71]. What is more, glatiramer acetate could perform a novel selective inhibitory role upon the production of DC-derived pro-inflammatory mediators (e.g., TNF and the major T_H_1 polarizing factor IL-12) and exert a facilitating role in DC-mediated induction of effector IL-4-producing T_H_2 cells as well as secretion of anti-inflammatory cytokines (e.g., IL-10, TGF-β), without affecting the maturation or immunostimulatory potential of DCs [72, 73]. In contrast to these findings, the inductive effect of glatiramer acetate on DC-derived IL-10 production rather than the inhibitory effect on DC-derived TNF-α production was observed upon stimulation [74]. Glatiramer acetate-mediated anti-inflammatory shift of DCs is considered to be related to the suppression of STAT1 signaling [73]. In addition, expression of co-stimulatory molecule CD40 on DCs from MS patients treated with glatiramer acetate was significantly reduced and correlated with the risk of recurrence [75]. However, the underlying mechanisms remain to be unraveled.

### Sphingosine 1-phosphate receptor (S1PR) modulators

Sphingosine 1-phosphate (S1P), a bioactive metabolic product of cytomembrane sphingolipids, acts as a key regulator of various physiological and pathophysiological processes, including cellular behaviors (e.g., survival, proliferation, adhesion and migration), vascular barrier function, and homeostasis, signaling via five distinct high-affinity G protein-coupled receptor (GPCR) subtypes, S1PR_1_ to S1PR_5_ [76]. Regulation of vascular barrier integrity and immune cell trafficking by S1P–S1PR signaling system is highly relevant to inflammatory processes, autoimmune response [77–80]. Importantly, the presence of S1PR_1_ is critical for the egress of lymphocytes from lymph nodes into lymph, a process mediated by the gradient of S1P concentration [81]. Till now, there have been four S1PR modulators (distinct receptor subtypes targeted) approved for treatment of MS [fingolimod (S1PR_1,3,4,5_), siponimod (S1PR_1,5_), ozanimod (S1PR_1,5_), and ponesimod (S1PR_1_)], and several therapeutic candidates currently in clinical development [e.g., etrasimod (S1PR_1,4,5_), ceralifimod (S1PR_1,5_), and amiselimod (S1PR_1,4,5_)] [76, 81]. Currently, most of published data on the role of S1PR modulators in the CNS come from in vivo and in vitro studies of fingolimod or siponimod.

The primary mechanism of effects of S1PR modulators has been considered to be selectively sequestering autoreactive lymphocytes within the lymph nodes and affecting T cell differentiation via binding to S1PR_1_ on lymphocytes, preventing the infiltration of autoreactive lymphocytes into inflamed CNS via binding to S1PR_1_ on endothelial cells and astrocytes [81], influencing microgliosis and astrogliosis [82, 83], as well as protecting against demyelination and promoting remyelination by interacting with S1PR_5_ on oligodendrocytes [84].

It is noteworthy that S1PR modulators are closely related to DC functions as well. First, DCs act as metabolic gatekeepers of thymic export in an S1P lyase (SPL)-dependent manner. Deleting SPL in DCs rather than thymic epithelial cells or other stromal cells could prevent T cell egressing from the thymus by disrupt the S1P gradient, which could be rescued by adoptive transfer of wild-type DCs [85]. Moreover, S1PR_1_-mediated T_H_17 differentiation is thought to be dependent on sustained S1PR_1_ signaling in myeloid cells rather than an intrinsic T cell effect [86]. Lack of S1PR_4_ expression on DCs could profoundly affect cytokine production and migration of DCs, and reduce T_H_17 polarization [87]. In addition, mature DCs generated in the presence of fingolimod showed an impaired phagocytic capacity and immunostimulatory property with down-regulated IL-1β, IL-6, IL-12, IL-23 and TNF-α, but up-regulated IL-10 secretion. T cells co-cultured with fingolimod-treated DCs were inhibited differentiating into pro-inflammatory T_H_1 cells or T_H_17 cells [88, 89]. Application of fingolimod in patients with MS could rebalance the immune tolerance networks by regulating the secretion of pro-inflammatory cytokines by APCs including DCs and monocytes, without a significant reduction in the absolute number of these cells [90].

Many studies have reported that this kind of medicines have the ability to regulate chemotaxis and trafficking of DCs. Administration of fingolimod to normal mice significantly decreased DCs in lymph nodes and spleen with down-regulated expression of CD11b, platelet endothelial cell adhesion molecule 1 (PECAM-1, CD31), intercellular cell adhesion molecule 1 (ICAM-1, CD54), and CCR7 on circulating DCs [91]. In vitro, trans-endothelial migratory capacity of fingolimod-treated immature DCs in response to the CCR7 ligand CCL19 was reduced [91]. Fingolimod could down-regulate the expression of CCR6 on immature DCs, inhibiting the migration of these cells [92]. Another in vitro study reported that fingolimod could reduce chemotaxis of both immature and mature DCs [88]. However, unimpaired migration of pDCs into the CNS could exert beneficial effects in the treatment of EAE with AUY954, an S1PR_1_-specific agonist [93].

In brief, S1PR modulators perform immunomodulatory roles through intricate mechanisms with sequestering autoreactive lymphocytes within the lymph nodes and influencing T cell differentiation, regulating the immunogenicity and trafficking of DCs, as well as cross-talks between these two aspects.

### Dimethyl fumarate

Dimethyl fumarate (DMF) and its active metabolite, monomethyl fumarate (MMF) function primarily by restricting the survival, proliferation, activation and cytokine secretion of specific effector and memory T cell subsets, thereby shifting the balance between T_H_1/T_H_17 and T_H_2 immune responses [94]. In addition, application of DMF has been shown to exert neuroprotective effects against oxidative stress for neurons, astrocytes and other CNS cells via activating the nuclear factor erythroid 2-related factor (Nrf2) signaling pathway [95].

Hydroxycarboxylic acid receptor 2 (HCAR_2_), a GPCR subtype, is a pleiotropically linked receptor for MMF that mediates the protective effects as well as inflammatory side effects via activating distinct pathways in different cell types [96, 97]. The effects of DMF/MMF on DCs mainly include inhibition of DC maturation, alteration of DC cytokine profile, and subsequently modulation of T_H_1/T_H_17 and T_H_2 cell differentiation balance, as well as induction of tolDCs [98–101]. It is well-known that antioxidant Nrf2 signaling plays a critical role in the underlying mechanism of effects of DMF/MMF on DCs [102]. Several studies indicated that alternative signaling pathways independent of Nrf2 are also involved with DMF effects on modulation of adaptive and innate immunity [98, 103, 104]. For example, the DMF-induced inhibition of NF-κB signaling pathway is thought to play an important role in the mechanism of DMF/MMF action on DCs, and can be further inhibited by DMF-mediated down-regulation of extracellular signal-regulated kinase (ERK)1/2 and its downstream kinase mitogen stress-activated kinase 1 (MSK1) [98]. Given that ERK activation has been demonstrated to be involved in CCL2-mediated DC transmigration across the BBB, DMF-mediated inhibition of ERK1/2 might contribute to the decrease of CNS recruitment of DCs which correlates with disease severity in EAE [45]. A recent study reported that DMF could modulate the functions of human pDCs through another mechanism of action independent of Nrf2. DMF could target the IL-1R-associated kinase 4 (IRAK4)–MyD88 signaling and inhibit IRAK4-mediated cytokine secretion in a cysteine 13-dependent manner, thereby disrupting human innate immune signaling [104]. Interestingly, in vitro experiments indicated that exposure of DCs to low doses of electrophilic Nrf2 activators including DMF could exert anti-inflammatory effects by inhibiting the production of pro-inflammatory cytokines (e.g., IL-1β and IL-12) in an Nrf2-dependent manner, while high doses of DMF might in contrast promote inflammatory apoptosis and concomitant IL-1β secretion independently of Nrf2 [105]. The exact mechanism by which DMF acts on DCs has not been fully elucidated, which need to be further explored as the pharmaceutical relevance in discovering novel molecular targets to optimize MS therapy.

### Teriflunomide

Teriflunomide, the active metabolite of leflunomide, exerts a cytostatic effect on lymphocyte proliferation by selectively, reversibly blocking dihydro-orotate dehydrogenase, which is a key mitochondrial enzyme for pyrimidine de novo biosynthesis [106, 107]. To date, there have been few reports on the effects of teriflunomide on DCs, mainly coming from other autoimmune diseases, such as rheumatoid arthritis, systemic lupus erythematosus, autoimmune uveitis, etc. Leflunomide, teriflunomide and the derivative (FK778) have been evidenced to inhibit DC maturation and migration, leading to an impaired potency to produce the pro-inflammatory cytokines/chemokines and to initiate proliferation and polarization of CD4^+^ T cells to T_H_1/T_H_17 cells [108–112]. These events are related to the suppression of NF-κB and activator protein-1 signaling pathways apart from the inhibition of pyrimidine synthesis [110–112].

### Monoclonal antibodies

Several monoclonal antibodies, such as alemtuzumab (anti-CD52), natalizumab (anti-α4 integrin), daclizumab (anti-CD25), ocrelizumab (anti-CD20), and ofatumumab (anti-CD20), most of which target to T or B cells, have been approved for MS therapy. There are evidences suggesting that these antibodies can impact DCs’ function.

Alemtuzumab (anti-CD52) can induce depletion and repopulation of T and B cells, also lead to a significant decline in the subsets of circulating DCs and a relative increase in the regulatory and memory DC phenotypes, promoting the restoration of tolerogenic networks [113–115]. In contrast, granulocyte–macrophage colony-stimulating factor (GM-CSF) and IL-23 production by DCs remained unaltered upon alemtuzumab application [114]. Most recently, Barbour et al. reported that anti-mouse CD52 monoclonal antibody could significantly ameliorate EAE severity, with increased expression of MHC-II and co-stimulatory molecules on peripheral DCs at first day post-injection and down-regulated level at 3 week post-injection [116].

Natalizumab, a recombinant humanized monoclonal antibody targeting the α4 subunit of α4β1- and α4β7-integrins that act as adhesion molecules in a wide range of human leukocytes including DCs, interferes with the migration of peripheral immune cells to the CNS [9, 117]. A detailed histological characterization found that the number of DCs, but not macrophages, microglia or T cells, within lesions decreased with the prolonged treatment duration [40]. Another study indicated that natalizumab could remarkably reduce the expression of MHC molecules and the numbers of DCs and CD4^+^ T cells in cerebral perivascular spaces of the patients with MS [118]. Moreover, DCs showed dose-dependent decrease of very late activation antigen 4 (VLA-4) expression levels and impaired capacity to prime antigen-specific T cell responses [119]. Notably, natalizumab therapy could enhance the expression of molecules with tolerogenic function including HLA-G, HLA-DR, PD-L1 and that of molecules with migratory function such as CCR7 on pDCs in patients with MS [120]. These findings suggest that DCs may be the primary effector cells of natalizumab therapy. The anti-inflammatory effects of natalizumab may be largely due to the reduced CNS infiltration of DCs and functional impairment of DC-T interactions as well as shift toward the tolerogenic phenotype of DCs.

Daclizumab is a humanized monoclonal antibody targeting IL-2 receptor α (IL-2Rα) chain (CD25) [121]. Antigen-activated DCs express IL-2Rα, representing an alternative cell type susceptible to daclizumab blockade. Given that the ability to secrete IL-2 endows mature DCs with unique T cell stimulatory capacity [122], daclizumab can block IL-2 trans-presentation by activated DCs to primed T cells, consequently restricting initial steps of antigen-specific T cell activation and expansion [123]. In addition, IL-2 signaling is critically required for the secondary expansion of CD8^+^ memory T cells [124]. Furthermore, daclizumab might shift the cytokine synthesis pattern of human DCs toward an anti-inflammatory T_H_2 profile in vitro [125]. Of note, however, IL-2 signaling is also critical for the development and function of CD4^+^CD25^+^FOXP3^+^ Tregs and the neutralization of IL-2 leads to induction of autoimmune diseases [126, 127].

### Mitoxantrone

Mitoxantrone is a chemotherapeutic agent approved for treatment of progressive and worsening MS based on its immunosuppressive properties. Mitoxantrone inserts itself within DNA strands, thereby disturbing the proliferation of various cells, particularly B cells, T cells, and macrophages. It has selective immune effects by inhibiting the production of pro-inflammatory cytokines, such as IFN-γ, TNF-α and IL-2 [128]. With regard to DCs, mitoxantrone can interfere with their antigen-presenting ability and induce programmed cell death or cell lysis [129]. Taken together, the effects of mitoxantrone on DCs are nonspecific.

### Cladribine

Cladribine (2-chlorodeoxyadenosine) is a synthetic purine nucleoside analog, resulting in a selective diminution in peripheral lymphocytes by disrupting DNA replication and repair [130]. In addition to the well-characterized cytotoxic activity toward lymphocytes, cladribine also exerts immunomodulatory effects on DCs. Apart from the capacity to induce apoptosis of DCs [131], cladribine can influence the maturation and T cell priming capacity of DCs, mediating T cell differentiation shift to T_H_2 polarization [132]. Interestingly, in vitro experiments showed that cladribine in therapeutic relevant concentrations could not induce the apoptosis of differentiated macrophages or DCs, nor could interfere with their phenotype [133], suggesting that cladribine does not impact the innate immune system under steady state conditions which is consistent with insight from clinical trials [134].

In short, current DMT drugs exert the direct or indirect effects on DCs, primarily influencing DC maturation and migration, resulting in an impaired potency to produce pro-inflammatory molecules and to initiate proliferation and polarization of CD4^+^ T cells, as well as reducing infiltration of inflammatory cells into the CNS. The underlying mechanisms might imply to search alternative novel targets for MS therapy.

## Inducing tolDCs for treatment in MS

Currently approved drugs for the treatment of MS have capacities to provide a remedy to alleviate symptoms via immunomodulatory role, rather than to provide a cure by directly addressing the immune tolerance invalidation. The inhibitory effects of these therapeutics on systemic inflammation may conduce to numerous adverse security risks, such as increased susceptibility to malignancies or opportunistic infections [25]. Novel immunotherapies are emerging to overcome these problems by achieving antigen-specific tolerance, which can suppress the pathogenic autoantigen induced autoimmune responses without compromising the protective immune responses.

Given the specialty of DCs to coordinate innate and adaptive immunity as well as the dual capacity to induce either inflammatory or tolerogenic responses, emerging therapies targeting or exploiting DCs may become a promising approach for MS treatment. DC-based therapy currently includes two major approaches [13]. One approach achieves global immune regulation by selectively targeting the dysregulated DC functions, such as maturation, antigen uptake, cytokine production or migration, or even by depleting DC subsets with key pathogenic roles. Another approach aims to restore the immune tolerance via DC-based autoantigen-specific therapeutic intervention by loading autoantigens to DCs [13]. Most of the current DMT drugs effect on DCs primarily via the former as described above.

Ex vivo tolDC induction strategies are typically based on reeducation of patient-derived DCs by various tolerance-inducing pharmacological agents (e.g., vitamin D3, aryl hydrocarbon receptor ligands, such as 2-(1′*H*-indole-3′-carbonyl)-thiazole-4-carboxylic acid methyl ester (ITE), and IL-27, etc.). The induced tolDCs will subsequently be reinfused into the body, presenting tolerogenic signals to T cells to suppress pathogenic autoimmune responses in a nonspecific manner [135–137]. Some tolDC-based vaccines for the treatment of MS have been shown to be safe and feasible in several phase I clinical trials [138, 139]. However, this kind of therapeutic strategies requires complicated handling and exorbitant expenditure. Due to the personalized nature of tolDCs generated in this way, its clinical application is limited.

In recent decades, in situ elicitation of a tolerogenic phenotype of DCs by specifically targeting varieties of cell-surface receptors has provided an alternative route to overcome these limitations and has been showing great promise in MS therapy, which will be discussed in detail in the following sections.

### In vivo induction of nonspecific tolDCs

Induction of nonspecific tolDCs in vivo via several cytokines or by activating aryl hydrocarbon receptors has been well-summarized by Takenaka and Quintana [26]. The immune checkpoints, such as PD-1/PD-L1 and CTLA-4 axis, are imperative for modulating the balance between immunity and tolerance.

PD-1 blockade or PD-L1 knockout could accelerate disease onset and exacerbate disease severity of EAE due to increased lymphocyte infiltration, antigen-specific T cell activation and cytokine production in the CNS [140, 141]. In contrast, up-regulation of PD-L1 on DCs via DNA demethylation could suppress EAE progression [2]. Signals delivered by PD-L1 via PD-L1-IgG2aFc fusion protein could exhibit a significant and long-lasting effect on disease severity by selectively interfering with T_H_17 responses [142]. Recently, we demonstrated that tolDCs induced by 1,25-dihydroxyvitamin D3 could exert therapeutic effects on EAE mice through the enhancement of PD-1/PD-L1 signaling pathway [143]. Therefore, the PD-1/PD-L1 axis may play a vital role in autoimmune conditions including MS/EAE and might be a promising target for novel immunotherapy.

CTLA-4 is capable to bind to CD80/CD86 molecules on DCs, transmitting negative co-stimulatory signals via trans-endocytosis of the B7-family molecules or regulation of tryptophan catabolism [144, 145]. Human DCs treated with CTLA4–IgG1Fc fusion protein are in an autophagy-deficient status with reduced immunogenic potential, probably due to the activation of the PI3K/Akt/mTOR pathway and FoxO1 nuclear exclusion and consequently down-regulated autophagosome formation [146]. Tregs could ameliorate autoimmunity by restraining DC function via CTLA-4-dependent down-regulation of autophagy [146]. These data suggest that CTLA-4 could mediate reverse signaling in DCs, which is beneficial for the control of autoimmune responses.

In addition, CD83 represents a promising immune checkpoint, which exists in a membrane-bound state (mCD83) or a soluble isoform (sCD83) [147]. Originally mCD83 as a marker for mature DCs has been shown to be widely distributed in a great variety of cell types (e.g., activated T and B lymphocytes, Tregs, as well as thymic epithelial cells) and to play vital roles in the orchestration of immunity and tolerance [147]. Several in vitro and in vivo studies have revealed that sCD83 has immunosuppressive properties by inducing tolDCs and suppressing DC-mediated T cell activation [148–153]. Strikingly, sCD83 treatment could significantly ameliorate the symptoms of EAE, with strongly reduced cytokine production, T cell proliferation, and leukocyte infiltration into the CNS [153]. Furthermore, DC-specific mCD83 deficiency could confer an overactivated DC phenotype, characterized by an enhanced capacity to induce antigen-specific T cell proliferation and T_H_17 differentiation, as well as to impair the suppressive function of Tregs, leading to dramatically aggravated immune responses in the EAE model due to the dysregulation of tolerance mechanisms [154]. These data provide new insights into the roles of CD83 in DCs and its therapeutic potential in MS/EAE.

### In vivo induction of autoantigen-specific tolDCs via targeting receptors on DCs

The effective therapeutic strategies designed for in vivo autoantigen-specific tolDC induction are supposed to deliver controlled amounts of disease-associated autoantigens into the antigen-processing and presentation procedures. Several strategies explored involves conjugating antigens with antibodies specific for the cell-surface receptors or with specific glycan structures that act as ligands for these receptors, as well as loading antigens into nanoparticles or liposomes [10, 11, 155]. Ideally, engineering materials should be uptaken by DCs without inducing a specific immune response, which can be achieved by co-delivery of tolerogenic agents (e.g., dexamethasone, rapamycin, IL-10, ITE and vitamin D3) together with disease antigens [156–161]. The most promising receptors currently being explored as potential feasible targets for in vivo tolDC generation will be discussed as the below (Table 2).

**Table 2:** Summary of strategies to induce autoantigen-specific tolDCs in vivo

Receptors	Animal model	Coupled antigen	Targeting strategy	Mechanisms	Ref.
DEC205	MOG-induced EAE	MOG	Anti-DEC205	Prevent accumulation of effector T cells Promote enrichment of anti-MOG transgenic T cells Induce tolerance by tuning T cell responses through CD5 induction	[165]
			Single-chain fragment variables specific for DEC205	Induce a suppressive phenotype of DCs that expresses PD-L1 and secretes IL-10 and TGF-β Induce activated, IL-10-producing CD4^+^CD25^+^ FOXP3^+^ Tregs Reduce T_H_1/T_H_17 cells	[166]
			Anti-DEC205	Expand and induce antigen-specific FOXP3^+^ T cells	[167]
	PLP-induced EAE	PLP	Anti-DEC205	Reduce T_H_17 cells Induce CD4^+^ FOXP3^+^ Tregs	[168]
MR	PLP-induced EAE	PLP	Mannosylation	Prevent CNS inflammation Induce a less-vigorous T_H_1 response Induce antigen-specific tolerance	[174–176]
Langerin	MOG-induced EAE	MOG	Anti-langerin	Expand and induce antigen-specific FOXP3^+^ T cells	[167]
DC-SIGN	–	–	Coupling anti-DC-SIGN to porous silicon rapamycin-loaded nanoparticles	Promote the generation of Tregs	[191]
DCIR	MOG-induced EAE	MOG	Anti-DCIR2	Expand and induce antigen-specific FOXP3^+^ T cells	[167]
	PLP-induced EAE	PLP	Anti-DCIR2	Induce deletion and/or anergy in PLP-reactive T_H_1/T_H_17 cells Enhance the antigen-specific suppressor activity of FOXP3^+^ Tregs	[196]
DNGR-1	MOG-induced EAE	–	A fusion of anti-DNGR-1-IFNQ124R	Not shown	[203]
MICL	MOG/PLP-induced EAE	–	Anti-MICL or MICL knockout	Reduce DC infiltration within CNS^a^	[206]
Siglec-H	MOG-induced EAE	MOG	Anti-Siglec-H	Inhibit CD4^+^ T cell expansion and T_H_1/T_H_17 cell polarization Fail to induce de novo generation of FOXP3^+^ Tregs	[211]
TLR9	MOG-induced EAE	–	Type A CpG ODNs	Reduce lymphocyte infiltration within CNS Induce tolerance phenotype of pDCs Inhibit T_H_1/T_H_17 immune response Induce expansion of Tregs and production of regulatory cytokines	[222]
	MOG/PLP-induced EAE	–	GpG ODNs	Reduce MHC-II expression Inhibit T_H_1 immune response Promote T_H_2 cell phenotype Reduce autoreactive B cell diversity	[223, 224]
	MOG-induced EAE	MOG	GpG-containing polyelectrolyte multilayers/polyplexes	Restrain TLR9 signaling and DC activation Inhibit T_H_1/T_H_17 immune response Induce CD4^+^CD25^+^ FOXP3^+^ Tregs	[226, 227]
MHC-II	MOG-induced EAE	MOG	Nanobodies recognizing MHC-II	Induce antigen-specific DC tolerance Elicit a burst of proliferation, followed by attrition, of MOG-specific CD4^+^ T cells	[228]

*CD* cluster of differentiation, *CpG* cytidine–phosphate–guanosine, *DC* dendritic cell, *DCIR* dendritic cell immunoreceptor, *DC-SIGN* dendritic cell-specific intercellular adhesion molecule-3-grabbing non-integrin, *DEC205* dendritic cell receptor for endocytosis-205, *DNGR-1* dendritic cell natural killer lectin group receptor-1, *EAE* experimental autoimmune encephalomyelitis, *IFN* interferon, *IL-10* interleukin-10, *MHC* major histocompatibility complex, *MICL* myeloid inhibitory C-type lectin receptor, *MOG* myelin oligodendrocyte glycoprotein, *MR* mannose receptor, *ODNs* oligodeoxynucleotides, *pDCs* plasmacytoid DCs, *PD-L1* programmed cell death ligand 1, *PLP* proteolipid protein, *Siglec-H* Sialic-acid binding immunoglobulin-type lectin-H, *TGF-β* transforming growth factor-β, *T*_*H*_ T helper cells, *TLR9* Toll-like receptor 9, *Tregs* regulatory T cells

^a^The MICL-mediated targeting DC-induced immune tolerance strategies remains in its infancy

### DC receptor for endocytosis-205 (DEC205)

One of the first and most frequently used receptors for in vivo targeting of DCs is DC receptor for endocytosis-205 (DEC205, also known as CD205). DEC205, a type I transmembrane C-type lectin receptor (CLR) homologous to the macrophage mannose receptor, is an endocytic receptor highly expressed by DC subsets [162]. DEC205-mediated endocytosis transfers captured antigens directly from the extracellular space to late endosomes or lysosomes rich in MHC molecules, entailing a greatly improved efficiency of antigen presentation [162, 163]. DEC205 provides an efficient receptor-based mechanism for DCs to process and present anti-DEC205 antibody-coupled antigens in vivo, resulting in peripheral T cell tolerance in the steady state or strong immunogenic responses in the presence of a maturation stimulus [163, 164]. Since then, DEC205 targeting has been explored in a variety of animal models of autoimmune diseases including EAE for MS. For example, several studies have demonstrated that delivery of autoantigen MOG peptide to DCs by anti-DEC205-MOG conjugates can lead to generation and expansion of IL-10-producing Tregs, deletion of MOG-specific T cells, down-regulation of T_H_1/T_H_17 cell activity, which can delay disease onset and ameliorate symptoms of EAE consequently [165–167], similar to the phenomena observed after anti-DEC205-mediated proteolipid protein (PLP) delivery to DCs in PLP-induced EAE [168]. Furthermore, it is interesting that migratory DCs induce MOG-specific Tregs more potently than lymphoid-resident DCs in the steady state [167]. In consequence, these data corroborate the potential of targeting DC via DEC205 to induce immune tolerance, protecting against MS and other autoimmune diseases.

However, unlike mouse DEC205 predominantly expressed by DCs, human DEC205 is expressed on more populations of leukocytes, including myeloid blood DCs and monocytes with relatively high levels of DEC205, B lymphocytes with moderate levels, and pDCs, T lymphocytes and NK cells with low levels [169]. Kato et al*.* reported that immature DCs expressed low levels of DEC205, which was significantly increased during activation, suggesting that DEC205 is an activation-associated molecule [170]. The broader expression pattern of human DEC205 could be an impediment for the development of clinical DEC205-mediated DC targeting strategies.

### Mannose receptor (MR)

The mannose receptor (MR, also known as CD206) is a type I transmembrane CLR mainly expressed by macrophages and DCs. In human DCs, MR has been demonstrated to mediate efficient presentation to T cells with high capacity and broad specificity receptors for glycosylated antigens (e.g., mannose, glucose, maltose, fucose and GlcNAc) [171]. In mice, the MR is expressed by a variety of cell types, including endothelial cells, perivascular microglia and mesangial cells, in addition to macrophages. MR can be detected in cultured moDCs; however, in situ expression of MR on murine DCs remains unknown [172]. In vitro experiments showed that application of a specific anti-MR monoclonal antibody PAM-1 and selected natural ligands such as biglycan and mannosylated lipoarabinomannan could result in an altered profile of cytokines/chemokines in moDCs with the ability to dampen the T_H_1 immune response and to favor the amplification of T_H_2 immune response [173]. A limited number of in vivo studies have shown that mannosylated myelin peptides could inhibit the development of EAE by inducing a state of immune tolerance primarily by acting on T cells [174–176]. The different expression patterns of MR would be an obstacle to fully replicating the receptor function in animal models and exploring the feasibility of MR-mediated DC targeting strategies.

### Langerin

Langerin (CD207), a type II membrane-associated C-type lectin, acts as an endocytic receptor on surfaces of Langerhans cells as well as DC subsets often co-expressing with DEC205 receptor, mediating efficient antigen presentation in vivo through capture and internalization of various ligands (e.g., fucose, mannose, n-acetylglucosamine, and sulfated sugars) [177]. Steady-state migratory RelB^+^langerin^+^ dermal DCs with a partially mature phenotype (MHC-II^int^CD86^int^CD40^hi^CCR7^+^), rather than epidermal Langerhans cells or lymphoid-resident DCs, can mediate peripheral induction and expansion of the antigen-specific Tregs in draining lymph nodes of mice using endogenous TGF-β [178]. In addition, lung langerin^+^ migratory DCs are capable to drive Treg cell differentiation [167]. In contrast, langerin^+^CD103^+^ DCs are superior to other DC subsets in stimulating myelin-reactive T cell proliferation and T_H_1/T_H_17 cell differentiation in a GM-CSF-dependent manner, while deletion of this DC subset in vivo confers resistance to EAE [179]. Delivery of autoantigen MOG peptide to skin and lung langerin^+^CD103^+^ migratory DCs by anti-langerin–MOG coupling can result in the generation and expansion of MOG-specific Tregs, consequently lessening symptom severity of EAE [167]. However, Flacher et al*.* reported that langerin^+^ dermal DCs targeting by anti-langerin–ovalbumin coupling could trigger long-lasting cytotoxic CD8^+^ T responses in the presence of additional adjuvants including the TLR3 ligand poly(I:C) and anti-CD40 agonist antibody, while Langerhans cells could induce cross-tolerance in similar conditions [180]. These data mark langerin as a promising target for in vivo delivery of self-antigens to DCs to protect against EAE; however, the effects can be affected by the sub-populations and mature status of DCs as well as the intensity of the activation signals.

Especially, in humans, langerin is expressed at low levels on DCs isolated from liver, lung, dermis and tissue-draining lymph nodes, in addition to being highly expressed on epidermal Langerhans cells [181]. Importantly, the expression of langerin on DC subsets in humans distinct from that in mice. Langerin is restricted to the CD1c^+^ DC subset (cDC2) in humans which is homologous to CD11b^+^ DCs in mice, while langerin is expressed by the XCR1^+^ DC subset (cDC1) of mice. Interestingly, langerin has not been identified on the freshly isolated CD1c^+^ blood DCs, but can be rapidly induced by TGF-β or serum via an activin receptor-like kinase 3-dependent pathway [181]. Therefore, langerin targeting strategies leads to different outcomes in the experiments with humans and mice, which may be problematic in the clinical translation of these strategies.

### DC-specific ICAM-3 grabbing non-integrin (DC-SIGN)

DC-specific ICAM-3 grabbing non-integrin (DC-SIGN) is a type II CLR, which is highly expressed on the surface of immature DCs but down-regulates upon maturation [182]. DC-SIGN is capable to recognize both exogenous ligands derived from various glycans-containing pathogens and endogenous glycoproteins (e.g., ICAM-3, ICAM-2, Mac-1, MOG, etc.) [183]. DC-SIGN was originally described as an adhesion molecule capable to bind ICAM-3 on naïve T cells with high affinity, facilitating initial DC-T interaction and DC-mediated T cell proliferation and activation [184]. DC-SIGN can bind to ICAM-2 on endothelial cells, regulating chemokine-induced trans-endothelial migration of DCs [185]. Its intracellular domain includes molecular motifs capable to activate Raf-1/NF-κB signaling pathway and subsequently regulates DC maturation [186]. Moreover, the internalization motifs of DC-SIGN are indicative of a role in antigen processing and presentation to T cells [187]. Hence, DC-SIGN is involved in regulating multiple aspects of immune function, including DC trafficking and maturation, antigen uptake and presentation, as well as DC-T interactions.

Interestingly, DC-SIGN contributes to pro-inflammatory (T_H_1/T_H_17) or anti-inflammatory (T_H_2) immune responses through the activation of distinct signaling cascades triggered by mannose or fucose, respectively. Mannose-rich antigens induce the recruitment of kinase Raf-1 via the DC-SIGN signalosome consisting of scaffold proteins LSP1, KSR1 and CNK, resulting in the acetylation of the NF-κB subunit p65 and thereby enhancing the production of pro-inflammatory cytokines to promote T_H_1/T_H_17 response [186, 188]. In contrast, fucose-rich antigens or fucosylated glycans favor adaptive T_H_2 immunity via activation of atypical NF-κB family member Bcl3 in an IKKε-CYLD-dependent way [189].

The relative specificity of DC-SIGN for DCs and its dynamic immunoregulatory roles endow DC-SIGN with potential as a molecular target to regulate the phenotype of immune response. Arosio et al*.* presented a potent DC-SIGN targeting antigen delivery device that developed using gold nanoparticles functionalized with α-fucosyl-β-alanyl amide, and with neutral effects toward DC maturation and IL-10 production [190]. When loaded with rapamycin, porous silicon nanoparticles targeting DC-SIGN could be taken up by splenic and peripheral blood DCs and enhance the generation of Tregs [191]. In consequence, DC-SIGN-mediated DC targeting strategy may be a feasible approach to induce immune tolerance in vivo.

Currently, however, DC-SIGN has rarely been explored as a DC target in MS/EAE, probably due to the difficulty of fully replicating its function in animal models. Further studies should focus on the homology of DC-SIGN receptor and its feasibility in DC-targeted immunotherapy.

### DC immunoreceptor (DCIR, CLEC4A)

DC immunoreceptor (DCIR), another member of type II CLRs, contains a carbohydrate recognition domain in its extracellular portion and an immunoreceptor tyrosine-based inhibition motif (ITIM) in the cytoplasmic portion. DCIR is expressed on various cell types including myeloid DCs, pDCs, immature and mature moDCs, macrophages, monocytes, B lymphocytes and neutrophils, and functions as an inhibitory receptor [192]. DCIR is considered as an APC receptor that is efficiently internalized into human moDCs in a clathrin-dependent manner upon triggering with anti-DCIR monoclonal antibodies. DCIR triggering can down-regulate TLR8-induced production of IL-12 and TNF-α, while do affect neither TLR4-/TLR8-mediated CD80 and CD86 up-regulation nor TLR2-/TLR3-/TLR4-mediated cytokine production [193]. Similarly, DCIR can be readily endocytosed into human pDCs in a clathrin-dependent manner upon receptor triggering. DCIR triggering can negatively affect TLR9-mediated IFN-α production while do not up-regulate the expression of co-stimulatory molecules. In turn, TLR9 triggering down-regulate the levels of DCIR on pDC maturation [194]. Furthermore, Dcir^−/−^ mice could develop exacerbated EAE with severe demyelination due to excess infiltration of DCs and T cells, indicating DCIR is a vital negative regulator of DC expansion [195]. Collectively, these data well elucidate the potential importance of DCIR in modulating DC function and DCIR/TLR cross-talk in maintaining the immune homeostasis. In addition, antigens targeted to DCs via DCIR are presented to T cells [194]. Therefore, delivery of autoantigen to DCs by targeting DCIR is a novel potential strategy to induce immunological tolerance.

Several studies have been conducted to investigate the potentials of DCIR targeting strategy in EAE treatment. Targeting DCs with anti-DCIR2-PLP_139–151_ fusion antibody could ameliorate EAE by deleting pathogenic T_H_1/T_H_17 cells as well as inducing expansion and activation of pre-formed Treg cells rather than inducing de novo generation of Tregs from naïve CD4^+^ T cell precursors [196]. However, antigen delivery with anti-DCIR2 was less efficient than anti-DEC205 or anti-langerin antibodies for the generation of MOG-specific Tregs, which was independent of administration route, time course, antibody dose, or antigen type [167]. Thus, DCIR2 may be a promising candidate for in vivo antigen delivery in mice, which needs more studies to validate the effectiveness and feasibility of DCIR2-mediated DC targeting in human settings.

### DC NK lectin group receptor-1 (DNGR-1, CLEC9A)

DC NK lectin group receptor-1 (DNGR-1, also known as CLEC9A), a type II transmembrane CLR, has attracted special attention due to its restricted expression pattern in human and mice DCs. DNGR-1 is selectively expressed by BDCA3^+^ myeloid DCs (cDC1s) in humans and by homologous CD8α^+^ DCs in mice, mediating endocytosis after sensing necrotic cells [197]. This receptor contains a cytoplasmic immunoreceptor tyrosine-based activation motif (ITAM)-like motif, which can recruit Syk kinase and induce the expression of pro-inflammatory cytokines [197, 198]. DNGR-1 triggering via anti-DNGR-1-antigen coupling can efficiently lead to receptor internalization and (cross-)presentation by human BDCA3^+^ myeloid DCs or murine CD8α^+^ DC subset to antigen-specific T cells, while anti-DNGR-1 antibody alone did not affect the maturation of DCs [199].

The restricted expression and endocytic property of DNGR-1 endow it potential as a promising DC target for in vivo antigen delivery. In addition, DNGR-1 activation can alleviate tissue damage-related immunopathology by dampening neutrophil recruitment [200]. In the steady state, administration of anti-DNGR-1-antigen conjugates could promote the differentiation into Tregs from naïve CD4^+^ T cells induced by CD8α^+^ DCs. However, co-administration with distinct adjuvants such as poly(I:C) and curdlan could prevent induced tolerance and drive T_H_1 and T_H_17 immune response, respectively [201]. Besides, targeting antigen to mouse DCs via DNGR-1 could induce strong CD4^+^ T cell responses biased toward a follicular helper phenotype [202]. Taken together, DNGR-1-mediated DC targeting strategies can induce tolerance or immunity depending on the type of antigen and adjuvant used. A novel protein-based DNGR-1 binding agent with a single domain nanobody has been shown to have DC targeting properties, exerting therapeutic effects on EAE when conjugated with IFNQ124R [203]. However, the sample size of this experiment was limited. The optimal conditions for tolerance induction via DNGR-1 targeting remain to be extensively studied.

### Myeloid inhibitory C-type lectin receptor (MICL, CLEC12A)

The myeloid inhibitory C-type lectin receptor (MICL, also known as CLEC12A, CD371) is another candidate DC-specific antigen targeting. Both murine and human MICL can selectively sense uric acid crystals, potentiating cell death-induced immunity under sterile conditions [204]. In mice, MICL is expressed mainly by CD8^+^ DCs and pDCs and by a small subset of CD8^−^ DCs as well as macrophages, monocytes and B lymphocytes. Similarly, MICL is expressed primarily by BDCA3^+^ DCs and pDCs as well as monocytes, macrophages and granulocytes in humans [205]. Its endocytic property has endowed this receptor with potential as an alternative DC target for antigen delivery. Delivery of ovalbumin to cDCs by conjugating to anti-MICL monoclonal antibodies could enhance antibody responses, while antibodies alone induced only moderate responses [205]. Interestingly, blockade of MICL with antibodies or MICL knockout might delay the onset and attenuate the severity of EAE by impairing DC binding and transmigration across the BBB [206]. However, the MICL-mediated targeting DC-induced immune tolerance strategies remains in its infancy.

### Sialic-acid binding immunoglobulin-type lectins (Siglecs)

Sialic-acid binding immunoglobulin-type lectins (Siglecs) are a family of immunomodulatory receptors expressed on a wide range of immune cells (NK cells, B and T lymphocytes, macrophages, microglia, monocytes, cDCs and pDCs, etc.) with different expressing patterns [207], enabling them to participate in diverse immune responses. In addition, the conserved Siglec-4, also known as myelin-associated glycoprotein, is expressed on oligodendrocytes and Schwann cells, playing a role in adhesion and signaling in glia–glia and/or axon-glia interactions and protecting neurons from acute toxic insults via a ganglioside-dependent mechanism [208]. The Siglec family functions via binding to sialic acids, which are present on the membrane of all living cells and act as a self-associated molecular pattern (SAMP) [207]. In the cytoplasmic domains, most Siglecs, such as Siglec-3 (CD33)-related Siglecs (e.g., Siglec-3, -5 till -12, -17, -E, -F and -G) and the conserved Siglec-2/4 carry regulatory motifs (e.g., ITIM, immunoreceptor tyrosine-based switch motif (ITSM), etc.), inducing inhibitory signaling and functioning as immune checkpoints to suppress unwanted immune responses through recruiting and activating Src homology 2 domain containing protein tyrosine phosphatase (SHP)-1 and SHP-2 [209]. Several other Siglecs (Siglec-14, -15, -16 and -H) possess amino acid residues in transmembrane domain which can correlate with activating the adaptor proteins, such as DNAX activation protein of 12 kDa (DAP12), an ITAM-bearing adaptor involved in cell activation [210]. Therefore, the Siglec family has the ability to suppress or augment immune responses.

The immunomodulatory capacity and endocytic property of Siglecs endows them with potential as targets in treatment of autoimmune diseases. In vivo experiments found that antigen (MOG peptides) delivery to murine pDCs by targeting Siglec-H could induce a decrease in CD4^+^ T cell expansion and T_H_1/T_H_17 cell polarization, without conversion to Tregs or deviation to T_H_2 cells, which could subsequently delay the onset and reduce the severity in EAE [211]. Likewise, both in vivo in EAE and in vitro, sialic acid-modified antigens targeting Siglec-E on DCs could induce an antigen-specific tolerogenic programming in DCs, dampening T_H_1/T_H_17 cell expansion and enhancing Tregs [212]. Taken together, targeting DCs through Siglecs might be a promising strategy to induce tolerogenic immune responses for treatment of MS. However, translation of research outcomes from animals to humans might be hampered by the ethnic heterogeneity of this receptor. Therefore, more preclinical studies are imperative to elucidate the feasibility.

### TLRs

TLRs are a collection of transmembrane proteins that induce overlapping yet distinct gene expression patterns, contributing to pro-inflammatory responses to fight infection/cancer [213, 214]. Notably, many TLRs, such as TLR2, TLR4, and TLR9, have been demonstrated to act as pivotal modulators of autoimmune process in MS/EAE [215–217].

TLR2 was reported to be expressed by oligodendrocytes and up-regulated in MS lesions, participating in the hyaluronan-mediated inhibition of oligodendrocyte precursor cell maturation and remyelination in a MyD88-dependent manner [215]. Interestingly, helminth antigens could exert strong regulatory effects on DCs and B cells via TLR2 in a MyD88-dependent or -independent manner [218]. A recent research reported that cell surface β-glucan polysaccharides of yeast could induce generation of Tregs from naïve T cells via a Dectin1–Cox2 signaling pathway in DCs and restrain T_H_1 polarization of effector T cells in a TLR2-dependent manner, thereby exerting powerful anti-inflammatory effects [219]. Thus, TLR2 may be a possible alternative DC target to modulate immune response. However, Shaw et al*.* proposed that receptor interacting protein 2 (RIP2), rather than TLR2, played a key role in the activation of CNS-infiltrating DCs [220]. In addition, the wide expression of TLR2 in other innate immune cells, such as macrophages, monocytes and microglia, also hampers the exploration of TLR2 targeting strategies.

With regard to TLR4, its triggering can induce different effects in different cell types. In mice deficiency of TLR4 solely in CD4^+^ T cells, the symptoms of EAE were almost completely abrogated, primarily due to the blunted T_H_1 and T_H_17 responses [216]. However, TLR-4 stimulation could activate DCs sufficiently to drive pathogenic T cell function in EAE [221]. Therefore, TLR4 has rarely been explored as a target for DCs.

TLR9, primarily expressed on B cells and pDCs and typically responsible for recognizing microbial cytidine–phosphate–guanosine (CpG) DNA, is up-regulated during EAE course [217]. It was reported that TLR9^−/−^ mice developed EAE with delayed severity compared with wild-type mice [217]. Excitingly, treatment with type A CpG oligodeoxynucleotides (ODNs) could effectively up-regulate Treg percentage in the spleen and inhibit T_H_1/T_H_17 immune response in the CNS, resulting in delayed onset of EAE, alleviated demyelination of the spinal cord and reduced severity of the disease. Those events were indicated to be mediated by type A CpG-induced tolerance phenotype of pDCs. Adoptive transfer of pDCs isolated from type A CpG-treated mice could also suppress CNS inflammation and attenuate EAE development [222]. Application of GpG ODNs, a TLR9 antagonistic ligand, could significantly suppress the activation of T_H_1 cells and induce a shift toward a protective T_H_2 immune response, alleviating the disease severity of EAE [223, 224]. Considering the intrinsic inflammation induced by delivery vehicles may exacerbate disease, several researchers have designed GpG ODNs self-assembled or co-assembled with MOG peptide into nanostructured polyelectrolyte multilayers/polyplexes to blunt TLR9 signaling to promote immunological tolerance [225–227]. Collectively, these data highlight the important role of TLR9 in driving MS/EAE and the potential of TLR9 targeting by specific ligands to induce immune tolerance and consequently alleviate the severity and progression of this disease. Future studies are needed to elucidate the uptake, trafficking and the underlying mechanisms of tolerance induction.

A most recent study reported that nanobody–autoantigen (MOG_35–55_) conjugates targeting MHC-II complexes on DCs could confer long-lasting protection against autoimmune conditions of EAE through tolerance induction, especially when co-administrated with the glucocorticoid dexamethasone via a cleavable linker [228]. These findings further support the therapeutic value of targeting DC-induced tolerance in MS treatment as well as the superiority of nanobodies for targeted delivery of small-molecule drugs or antigenic peptides. Collectively, the specific antigen delivery via DC targeting strategies mediated by various surface receptors may be promising to induce immune tolerance, which needs to be further explored.

### In vivo induction of autoantigen-specific tolDCs via non-inflammatory mRNA vaccines

The ideal strategy for treating autoimmune disorders is to generate autoantigen-specific tolerance in a non-inflammatory setting without suppressing the normal immune response. It is worth noting that in vivo DC-modifying strategies via targeting surface receptors have limitations due to the complexity of receptor expression profiles and overlapping signaling pathways. It is exciting that Ugur Sahin and colleagues has formulated an mRNA vaccine consisting of a lipid nanoparticle packed with a modified mRNA encoding MOG_35–55_, representing a novel tolDC-inducing approach [229]. The modification, replacing uracil with 1-methylpseudouridine (m1Ψ), aimed to abrogate the activation of TLR signaling and subsequent strong T_H_1 responses [230, 231]. In mice with MOG_35–55_-induced EAE, the m1Ψ mRNA vaccine could allow in vivo delivery of MOG peptides into DCs in a non-inflammatory context and induce MOG-specific tolerance, thereby alleviating the demyelination of the spinal cord as well as the clinical symptoms [229]. Thus, the m1Ψ mRNA vaccine encoding specific autoantigens is a promising approach to confer bystander tolerance and enable control of multiple autoimmune disorders including MS/EAE.

## Conclusions

Currently approved drugs for the treatment of MS provide a remedy to alleviate symptoms via immunomodulatory effects. In recent decades, reshaping immune balance achieved through inducing immune tolerance has proven to be a promising strategy for MS therapy. The unique ability of DCs to coordinate innate and adaptive immunity as well as the dual capacity to induce either immunity or tolerance makes DCs as an attractive target. Several surface receptors on DCs have been explored as promising targets to induce DC-mediated immune tolerance via delivery of specific autoantigens. Nanobodies or nanostructured polyelectrolyte multilayers have shown great advantages and prospects as delivery vehicles. However, the complexity of receptor expression profiles on various immune cells and overlapping signaling pathways have probably limited the development of this therapeutic strategy. Micro-environmental factors are also involved in modulating the immune effects of receptor-mediated DC-targeting strategies. In addition, incompatibilities between DCs in mice and human beings still hamper the clinical translation of research findings. Strikingly, selective delivery of autoantigens into DCs via non-inflammatory mRNA vaccines is an effective way to induce and maintain natural immune tolerance, promising for the treatment of a variety of autoimmune diseases. Further studies are supposed to identify the molecular mechanisms responsible for the tolerogenic phenotype of DCs to guide feasible immunotherapeutic strategies.

## Data Availability

Not applicable.

## Abbreviations

APCs: Antigen-presenting cells
BBB: Blood–brain barrier
CD: Cluster of differentiation
cDC1: Type 1 conventional DC subset
cDC2: Type 2 conventional DC subset
CLR: C-type lectin receptor
CNS: Central nervous system
CpG: Cytidine–phosphate–guanosine
CTLA-4: Cytotoxic T-lymphocyte antigen 4
DAP12: DNAX activation protein of 12 kDa
DCIR: DC immunoreceptor
DC-SIGN: DC-specific ICAM-3 grabbing non-integrin
DCs: Dendritic cells
DEC205: DC receptor for endocytosis-205
DMF: Dimethyl fumarate
DMT: Disease-modifying therapies
DNGR-1: DC NK lectin group receptor-1
EAE: Experimental autoimmune encephalomyelitis
ERK: Extracellular signal-regulated kinase
Flt-3L: FMS-like tyrosine kinase 3 ligand
GM-CSF: Granulocyte–macrophage colony-stimulating factor
GPCR: G protein-coupled receptor
HCAR_2_: Hydroxycarboxylic acid receptor 2
ICAM: Intercellular cell adhesion molecule
IDO: Indoleamine 2,3-dioxygenase
IFN: Interferon
IL: Interleukin
IRAK4: IL-1R-associated kinase 4
ITAM: Immunoreceptor tyrosine-based activation motif
ITE: 2-(1′*H*-Indole-3′-carbonyl)-thiazole-4-carboxylic acid methyl ester
ITIM: Immunoreceptor tyrosine-based inhibition motif
ITSM: Immunoreceptor tyrosine-based switch motif
LPS: Lipopolysaccharide
MBP: Myelin basic protein
mCD83: Membrane-bound CD83
MHC: Major histocompatibility complex
MICL: Myeloid inhibitory C-type lectin receptor
MMF: Monomethyl fumarate
MMP: Matrix metalloproteinase
MR: Mannose receptor
MSK1: Mitogen stress-activated kinase 1
Nrf2: Nuclear factor erythroid 2-related factor
moDC: Monocyte-derived DC subset
MOG: Myelin oligodendrocyte glycoprotein
MS: Multiple sclerosis
m1Ψ: 1-Methylpseudouridine
NADPH: Nicotinamide adenine dinucleotide phosphate
NF-κB: Nuclear transcription factor-κB
NK: Natural killer
ODNs: Oligodeoxynucleotides
pDC: Plasmacytoid DC subset
PD-L: Programmed cell death ligand
PECAM-1: Platelet endothelial cell adhesion molecule 1
PLP: Proteolipid protein
RIP2: Receptor interacting protein 2
RRMS: Relapsing–remitting MS
SAMP: Self-associated molecular pattern
sCD83: Soluble CD83
SHP: Src homology 2 domain containing protein tyrosine phosphatase
Siglecs: Sialic-acid binding immunoglobulin-type lectins
SPMS: Secondary-progressive MS
STAT: Signal transducer and activator of transcription
S1P: Sphingosine 1-phosphate
S1PR: Sphingosine 1-phosphate receptor
SPL: S1P lyase
TCRs: T cell antigen receptors
TGF: Transforming growth factor
T_H_: T helper cells
TLR: Toll-like receptor
TNF: Tumor necrosis factor
tolDCs: Tolerogenic DCs
TRAIL: TNF-related apoptosis-inducing ligand
Tregs: Regulatory T cells
VLA-4: Very late activation antigen 4
